# Comparison of the effectiveness of lecture instruction and virtual reality-based serious gaming instruction on the medical students’ learning outcome about approach to coma

**DOI:** 10.1186/s12909-021-02771-z

**Published:** 2021-06-15

**Authors:** Meysam Siyah Mansoory, Mohammad Rasool Khazaei, Seyyed Mohsen Azizi, Elham Niromand

**Affiliations:** 1 Department of Biomedical Engineering, School of Medicine, Kermanshah University of Medical Sciences, Kermanshah, Iran; 2 Fertility and Infertility Research Center, Health Technology Institute, Kermanshah University of Medical Sciences, Kermanshah, Iran; 3 Medical Education and Development Center, Arak University of Medical Sciences, Arak, Iran; 4 Fertility and Infertility Research Center, Health Technology Institute, Kermanshah University of Medical Sciences, Kermanshah, Iran

**Keywords:** Virtual reality, Serious game, Lecture, Coma, Medical students

## Abstract

**Background:**

New approaches to e-learning and the use of virtual reality technology and serious game in medical education are on the rise. Therefore, the purpose of this study was to compare the effectiveness of lecture method and virtual reality-based serious gaming (VRBSG) method on students learning outcomes about the approach to coma.

**Methods:**

We adopted a randomized trial method for this study and selected 50 medical students dividing them into experimental and control groups. Students’ learning outcome was measured with a 10-item test. Serious game usability scale was used to evaluate the usability of the serious game. Descriptive and inferential statistics were used for data analysis by SPSS-22 software.

**Results:**

Students’ familiarity with e-learning and VRBSG was low. The mean usability of a VRBSG was 126.78 ± 10.34 out of 150. The majority of students were eager to be instructed through VRBSG. The mean score of learning outcomes in the experimental group was significantly higher than the control group (t = − 2.457, *P* = 0.019).

**Conclusion:**

Students’ learning outcomes in the VRBSG group in the test approach to coma were significantly better than the lecture group. The usability of the serious game instruction method was high. Taken together, instruction through VRBSG had an effective role in medical students’ learning.

## Background

E-learning is one of the most important phenomena in the digital age [1, 2]. One of the emerging forms of information and communication technology (ICT) is virtual reality which has many applications in e-learning [3]. It produces artificial or real three-dimensional (3D) multimedia environment, which are created by modern computers. Based on VR technology, the user can discover or manipulate these environments [4, 5]. In recent years, the use of VR technology in the health professional education has been considered [3, 6, 7].

Empirical evidence indicates the positive effect of this technology on increasing the quality of learning, clinical competence, knowledge and skills of students [4, 8] and their empathy with patients [9]. In addition to VR technology, a new instruction method that has been emphasized in many studies is the serious game (SG) [10–13]. Serious games have educational purposes and are designed using digital technologies.

The substantial benefits of SG in medical education include active learning, reducing clinical problems, enhancing experience in low-risk environments [14, 15], enhancing analytical thinking, and enhancing communication levels in learning [16–18]. The results of some studies show that the combination of VR technology and SG method can increase the level of satisfaction with the learning process [16]. virtual reality-based serious gaming (VRBSG) is more effective than traditional instruction [4, 16]. A review of the literature showed that VR and SG have the high potential to enhance the quality of medical education and reduce some of the challenges in the conventional instruction [4].

One of the most important reasons for the present study is that limited studies have been conducted to evaluate the effectiveness of VRBSG in medical education. Therefore, there is a need for further studies in this area. Furthermore, there are many challenges in teaching the approach to coma in the hospital environment using traditional teaching method such as a larger number of students, special conditions in different wards of the hospital, and the importance of time management for the treatment of coma patients.

Today, the development of professional competencies in health science students and the establishment of a link between theoretical and practical expertise is one of the most important concerns in medical universities around the world [8]. The use of passive teaching methods such as lecturing does not have enough potential to achieve these goals. Therefore, it seems that the use of virtual reality technology and new approaches to education such as serious games is a very good solution to achieve the goals of medical education.

Therefore, the purpose of this study was to compare the effectiveness of lecture method and VRBSG method on Iranian students’ learning outcomes about the approach to coma. In this regard, we intend to answer the following hypotheses:

H1: significant difference between the effectiveness of education through VRBSG method and lecture method on learning outcomes of medical students.

H2: the usability of education through VRBSG is at an acceptable level.

## Methods

This study was performed using a randomized trial pretest-posttest design with a control group in Kermanshah, Iran in 2019. The randomized trial method is an interventional analytical study that is the most appropriate method to compare the effectiveness of a method [19]. The present study is based on the STROB checklist.

### Study sample

The sample size was calculated based on the statistical indicators obtained in Aqel and Muayyad study and based on the formula for calculating the sample size and 16 people with 10% sample loss. Therefore, in order to increase the accuracy of the findings, the sample size was increased to 50 people.

$$ N=2\times \left({\left({Z}_{1-\left(a\right./\left.2\right)}+{Z}_{1-\beta}\right)}^2\times \left.\Big(S{D}_1^2+S{D}_2^2\right)\right)/{\left({\overline{X}}_1-{\overline{X}}_2\right)}^2 $$

A total of 50 medical internship students voluntarily participated in this study, which was divided randomly into two groups (each group consisting of 25 students). Inclusion criteria were studying at the internship course and willingness to participate in the study. Unwillingness to participate in the study, failure to participate in the test approach to coma, and failure to respond to the serious game usability scale were the exclusion criteria.

### Data collection instrument

Data collection instruments included test and serious game usability scale (SGUS). A final test was given to evaluate the students’ learning outcomes about approach to coma; it had 10 questions for each of which there was a correct answer. The score range was from 0 to 20. The final test questions to assess students’ learning products were related to the level of knowledge. Also, the type of final exam questions was multiple choices. The high score indicates more knowledge of the approach to coma. The face and content validity of this tool was confirmed by five physicians (physicians specializing in Neurology and Neurosurgery). Cronbach’s alpha coefficient of 0.895 was used to determine the internal consistency of the test.

The level of the usability of the serious game instruction method was evaluated by the researcher-made scale of serious game usability. The first part of the scale included demographic questions. SGUS consisted of 30 items and four sub-scales of satisfaction, efficiency, helpfulness and learn ability. All items were rated on a 5-point Likert scale measure from strongly disagree [1] to strongly agree [5]. The overall range of scale scores was from 30 to 150.

Content Validity Index (CVI) and Content Validity Ratio (CVR) were used to evaluate the validity of the questionnaire. To determine the content validity ratio, a questionnaire was given to 10 specialists in educational technology and medical education and they were asked to evaluate the necessity of each item. Also, in order to check the content validity index, experts were asked to check the items in terms of relevance to the purpose of the study, clarity and simplicity of each item. After reviewing the views of experts, CVI and CVR indices were calculated at the level of 0.80. Therefore, based on these indicators, the questionnaire had an acceptable validity. In order to evaluate the internal consistency of the questionnaire, Cronbach’s alpha coefficient method was used and its reliability was calculated to be 0.86.

### Procedure

The implementation period in this study was from July to November 2019. Initially, the study permission was obtained from the National Ethics Committee of Kermanshah University of Medical Sciences. Internship medical students were invited to participate in the present study by announcing in the classroom. The main purpose of the study was explained to the samples. Students who were willing to participate in the study entered the study and were randomly divided to two groups (Experimental and control groups).

**Fig. 1:**
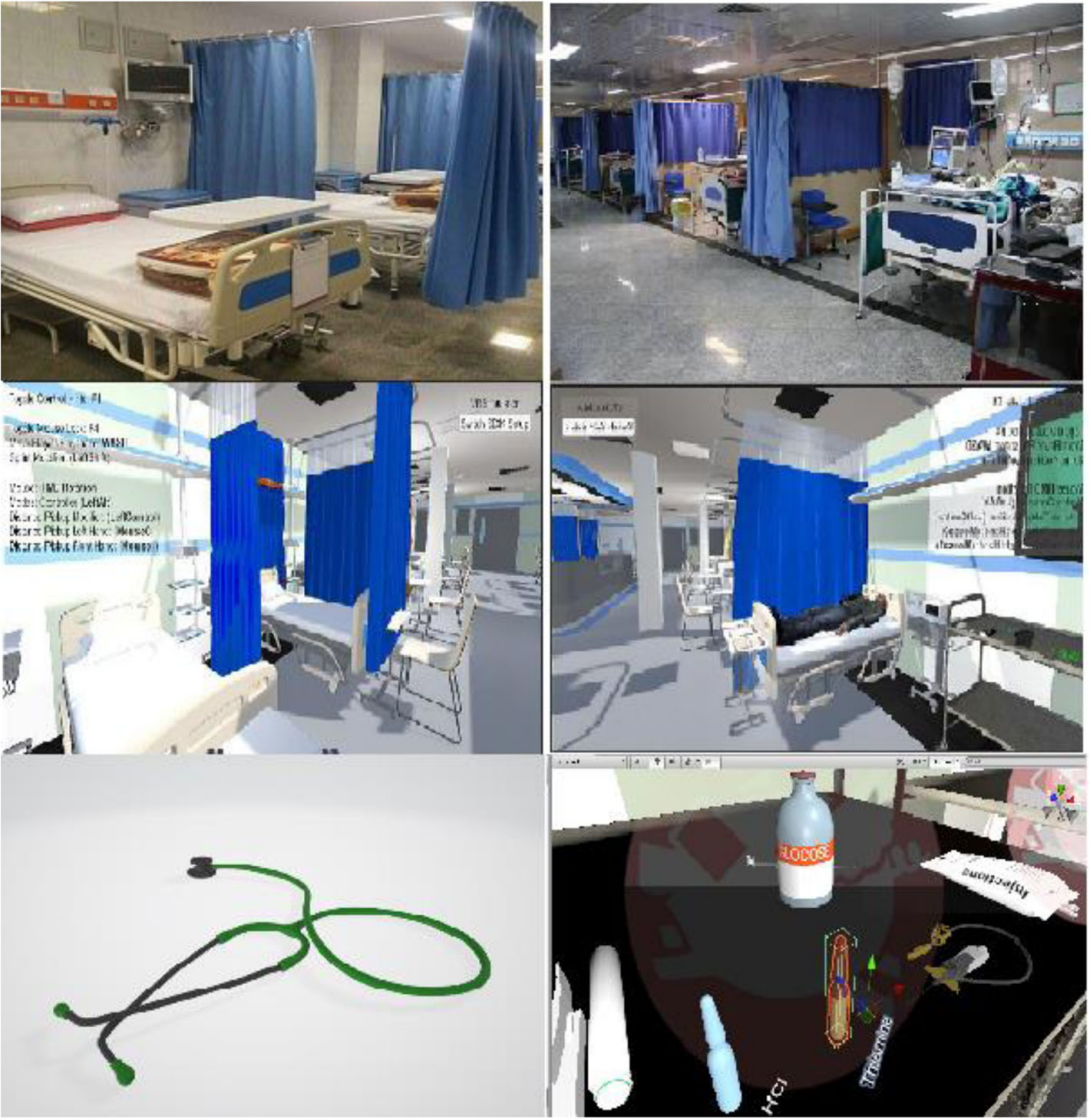
Real and simulated emergency department and game elements

An instruction session was held for both groups to learn about approach to coma. Harrison’s Manual of Medicine was used to teach the approach to coma. The teaching method for the control group was a lecture and each session it lasted 30 min. The teaching method for the experimental group was VRBSG. At first, the hospital environment was filmed to design the 3D game environment. Next, the game elements were selected and simulated. In order to reinforce the feeling of real presence and immerse the students, we tried to design the game environment and game elements similar to the real environment as shown in Fig. 1.

In addition, we increased the speed of interacting with the game and receiving feedback from the game. A very important advantage in serious game is its ability to increase students’ interaction in the learning process and their motivation to learn. That’s why we focused on this feature of the game and increased the amount and speed of interaction.

**Fig. 2:**
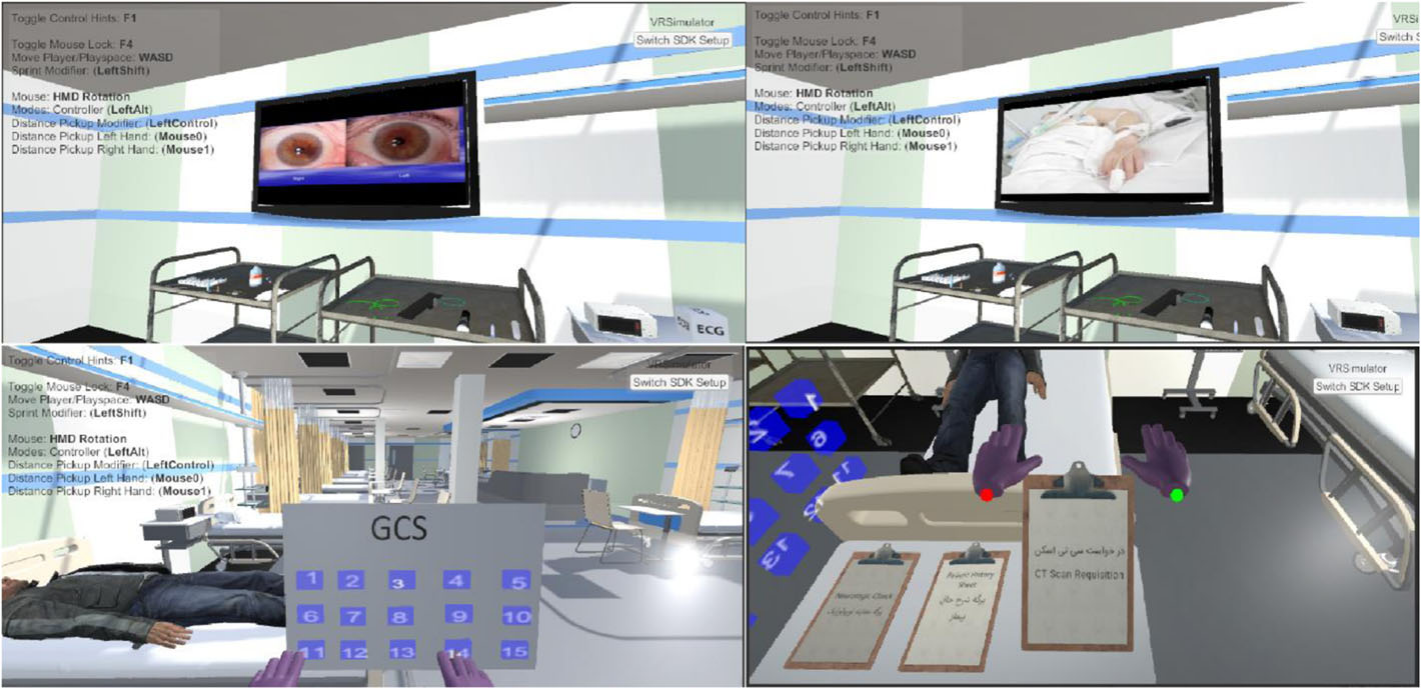
A few screenshots of the VRBSG environment

The game stages were designed by Unity game engine, ZBrush, Blender, V-Ray and 3ds Max software. During the game, the student had to provide initial diagnosis and treatment to the virtual patient. During the game stages, the student could make only three mistakes. If a student successfully completed all the stages of the game, they would receive a reward through the game. A few screenshots of the VR-based serious gaming environment are shown in Fig. 2. A final exam was given to evaluate students’ learning outcomes in both groups. The usability of the VRBSG method was evaluated from the viewpoint of the experimental group students. After completing the steps, data were collected and analyzed.

### Medical curriculum in Iran

The medical curriculum in Iran has been developed using a systematic planning strategy and taking into account the expected competencies (Competency oriented). In presenting this curriculum, it is possible to use various educational strategies including Problem based education, hospital based education, and subject based education. The stages of the Doctor of Medicine course in Iran include 4 stages of basic sciences, clinical preparation, pre-internship and internship. The duration of the 7-year course is 293 credits.

### Ethical consideration

The study was approved by the National Ethics Committee of KUMS (IR.KUMS.REC.1398.1194). In the present study, the ethical standards of the Helsinki Declaration have been respected. Written informed consent was obtained from all of the students. Test scores and demographic information of the samples were confidential.

### Statistical analysis

Data were analyzed by IBM SPSS Statistics v.22.0 software. The analysis was performed using descriptive and inferential statistics. According to the Kolmogorov-Smirnov test, the distribution of data was normal. Therefore, paired t-test and independent t-test were run to compare the mean scores of the experimental and control groups. Significance level was set at *p* < 0.05.

## Results

Of the 50 medical students participating in this study, 56.0% were male and 44.0% were female. The mean age of students was 24.30 ± 1.66. The majority of students were in the age range of 23 to 25 years (*n* = 29, 58%). The greater part of students had limited familiarity with e-learning (56%) and VRBSG (60%). As much as 70% of students tended to be instructed through VRBSG (Table 1). Mean and standard deviation score of VRBSG usability was 126.78 ± 10.34 out of 150.

**Table 1:** Demographic information and students’ views on new educational technologies

variable		Frequency (%)
Sex	Male	28 (56.0%)
	Female	22 (44.0%)
Age group	20–22	10 (20.0%)
	23–25	29 (58.0%)
	26≤	11 (22.0%)
Familiarity with e-learning system	Low	28 (56.0%)
	Moderate	15 (30.0%)
	High	7 (14.0%)
Familiarity with virtual reality-based serious game	Low	30 (60.0%)
	Moderate	16 (32.0%)
	High	4 (8.0%)
Willingness to teaching by serious game	Low	5 (10.0%)
	Moderate	10 (20.0%)
	High	35 (70%)

**Fig. 3:**
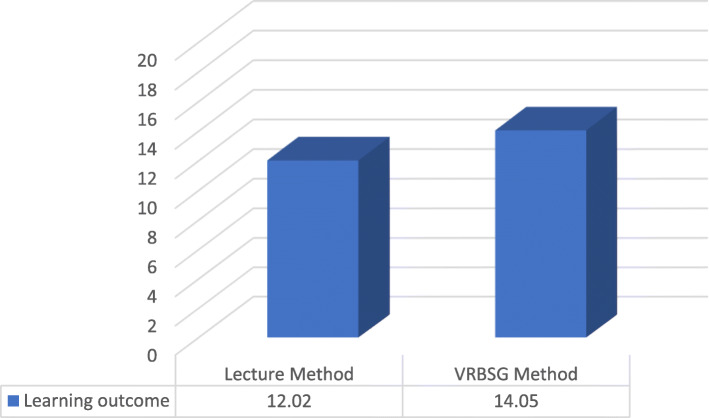
Comparison of the mean score of students’ learning outcome in the two groups

In Table 2, the results of the paired t-test are presented. Mean score of the learning outcome in the lecture group were 8.50 ± 1.48 and 12.02 ± 3.45, respectively. There was a significant difference between the pre-test and post-test (t = − 6.846, *P* = 0.001). The mean scores of the learning outcome in VRBSG group were 8.35 ± 1.49 and 14.05 ± 1.27, respectively. In this group, the difference between pre-test and post-test scores was statistically significant (t = − 13.357, *P* = 0.001) (Table 2). The result of the independent t-test is presented in Table 3. The mean score of learning outcome was higher in the VRBSG group (14.05 ± 1.27) compared to that in the lecture group (12.02 ± 3.45) (Fig. 3). According to Levene’s test (*F* = 21.669, *P* = 0.001) and t-test (t = − 2.457, *P* = 0.019), this difference was statistically significant (Table 3).

**Table 2:** Statistical analysis of pretest and posttest results of the Lecture and VRSG groups

Group	Pretest	Posttest	t	*P*-value
	Mean (SD)	Mean (SD)		
Lecture instruction	8.50 ± 1.48	12.02 ± 3.45	−6.846	0.000
VRSG instruction	8.35 ± 1.49	14.05 ± 1.27	−13.357	0.000

**Table 3:** Comparison of mean scores of students’ learning outcome in lecture instruction and VRSG instruction

Variable	Lecture instruction	VRSG instruction	Mean Difference	Levenes Test for Equality of Variances		t	*P*-value
	Mean (SD)	Mean (SD)		F	*P*-value		
Level of knowledge about approach to coma	12.02 ± 3.45	14.05 ± 1.27	−2.02	21.669	0.000	−2.457	0.019

## Discussion

The current study was conducted with the purpose of comparing the effectiveness of lecture method and VRBSG method on students’ learning outcomes about approach to coma. The results of first hypothesis showed that the scores of learning outcome in the VRBSG group were higher than the lecture group. Based on this, the first hypothesis of the study was confirmed.

This finding is consistent with the findings of some studies. In this regard, Aksoy conducted a study in the effects on the learning outcomes of tablet-based and VRBSG modules for basic life support training. The results of the study indicated that both modules of serious game affect students’ learning level. However, VRBSG was more effective [11]. Middeke et al. conducted a study on the effect of the serious game and problem-based learning on clinical reasoning. The result from their study showed that the score of clinical reasoning in the SG group was higher than the problem-based learning group [20]. A study was conducted at the Freiburg University Medical Center in Germany. Based on the results of this study, Urinalysis training using game-based e-Learning is more effective than the conventional instructional method [13]. The results from another study showed that simulated games had a significant effect on students’ motivation and cognitive skills [21]. Based on the results of the second hypothesis test; VRBSG usability is high and acceptable.

In this regard, the results of a systematic review showed that serious game has a great potential in health education. According to this systematic review, the results of 14 studies have shown that students’ academic performance in the serious game instruction method is better than the traditional instruction method [17]. Based on the results of the present study and the evidence obtained from the studies, we believe that considering the importance of using flexible education models in medical education, the application of VRBSG method can be an important step towards increasing the quality of the learning-teaching process [21]. The use of serious game technology as an educational method can play an important role in promoting knowledge, skills, motivation and participation of medical students.

Taken together, we believe that the new methods of e-learning such as VRBSG can have an effective role in enhancing the quality of medical education. Attractiveness is an important feature of the game. In terms of education, SG for students should be attractive and engaging. Designers must have the knowledge and skills to design a serious game in medical education. They should also be familiar with the principles of educational design. The provision of standard technological infrastructure in medical universities is important for the use of the serious game in the education process [22].

Examining the dimensions of the present study with other studies shows that, like this study, in other studies, the effectiveness of the two teaching methods on students’ learning outcomes has been compared. In some studies, two technology-based approaches have been compared. But in some other studies, a conventional instructional method has been compared to a newer method in e-learning. Another similarity between the present study and other studies in the field of virtual reality and serious gaming was the lack of familiarity of students with the use of this technology. In Iran, as a developing country, very few universities are familiar with virtual reality technology. Therefore, it is necessary to provide conditions for providing training to familiarize students and faculty members with virtual reality technology and a serious game approach.

Few studies have been performed on the effectiveness of VRBSG. The use of virtual reality technology and serious play in medical education is also a new approach. Therefore, it is necessary to conduct further studies in order to accurately identify its educational applications, how to integrate it in the teaching-learning process, increase the knowledge and awareness of students and faculty members about it and understand the advantages and disadvantages of using it in clinical education.

This study had some limitations. The first limitation was the duration of the samples’ cooperation. Students participating in this study were working in different wards of the hospital, so they participated in the study process for less than an hour in each session. Another limitation in the present study was the challenge of financing as virtual reality software and related technologies in Iran are very expensive. The third limitation was the data collection tool. A self-reporting scale was employed to assess the usability of VRBSG, which might have affected the accuracy of the results. We attempted to reduce this limitation by reassuring the students about the confidentiality of their information and responses. In the present study, students exhibited limited background information and familiarity with serious gaming, virtual reality, and e-learning. Taken together, our study showed that VRBSG, as a complementary teaching method, is an effective method to increase students’ learning.

## Conclusion

The present study was the first study on the effectiveness of VRBSG method on Iranian students’ learning outcomes about the approach to coma. The most important finding of this study was that the score of learning outcome about approach to coma was higher in the VRBSG group than in the lecture group. In this study, medical students tended for an instruction through a serious game. In addition, from their point of view, the usability of VRBSG was high. We believe that students’ readiness and acceptance of the instruction method through a serious game plays a key role in the successful use of this method. Providing pedagogical and technological infrastructures in medical universities is essential for implementing new approaches to e-learning.

## Data Availability

Data and materials are available by contacting the corresponding author.

## Abbreviations

VRBSG: Virtual reality-based serious gaming
SPSS: Statistical package for social sciences
ICT: Information and communication technology
3D: three-dimensional
KUMS: Kermanshah university medical sciences
SGUS: Serious game usability scale
